# Access to and use gaps of insecticide-treated nets among communities in Jimma Zone, southwestern Ethiopia: baseline results from malaria education interventions

**DOI:** 10.1186/s12889-015-2677-2

**Published:** 2015-12-29

**Authors:** Zewdie Birhanu, Lakew Abebe, Morankar Sudhakar, Gunawardena Dissanayake, Yemane Yihdego, Guda Alemayehu, Delenasaw Yewhalaw

**Affiliations:** Department of Health Education and Behavioral Sciences, Jimma University, Jimma, Ethiopia; President’s Malaria Initiative, United States Agency for International Development, Addis Ababa, Ethiopia; Abt Associates African Indoor Residual Project, Addis Ababa, Ethiopia; Department of Medical Laboratory Sciences, Jimma University, Jimma, Ethiopia

**Keywords:** Long lasting insecticide treated net, LLIN ownership, LLIN use, LLIN access, Behavioral change communication, Malaria, Ethiopia

## Abstract

**Background:**

Malaria remains one of the major public health concerns in Ethiopia. Use of long- lasting insecticidal nets (LLINs) is the country’s key malaria prevention and control strategy. This study intended to determine access to and usage gap of LLINs in malaria endemic settings in Southwestern Ethiopia.

**Methods:**

Data were collected from 798 households in three districts (Mana, Kersa and Goma) of Jimma Zone, Southwestern Ethiopia, from December 2013 to January 2014. The data were analyzed using SPSS software package version 17.0. LLINs ownership, access and utilization gap were determined following the procedure developed by Survey and Indicator Task Force of the Roll Back Malaria Monitoring and Evaluation Reference Group. To complement the quantitative data, focus group discussions and interviews were conducted with community groups and key informants.

**Results:**

In this study, 70.9 % (95 % CI: 67.8–74.1 %) of the surveyed households had at least one LLIN, and 63.0 % (95 % CI: 59.6–66.3 %) had sufficient LLINs for every member of the household. With respect to access, 51.9 % (95 % CI: 50.5–53.5 %) of the population had access to LLIN. Only, 38.4 % (95 % CI: 36.9–39.9 %) had slept under LLIN the previous night with females and children having priority to sleep under LLIN. This gave an overall use to access ratio of 70.2 % which resulted in behavior-driven failure of 29.8 %. Of the households with sufficient LLIN access, females (AOR = 1.52; 95 % CI:1.25–1.83; *P* = 0.001) and children aged 0–4 years (AOR = 2.28; 95 % CI:1.47–3.53;*P* = 0.001) were more likely to use LLINs than other household members. Shape of nets, sleeping arrangements, low risk perception, saving nets for future use, awareness and negligence, and perception of low efficacy of the LLINs contributed to behavioral failures.

**Conclusions:**

LLIN use was hampered by lack of ownership and most importantly by behavioral driven gaps. This calls for designing and implementing appropriate behavioral change communication strategies to address behavioral failure. Improving access to LLINs also needs attention. Further, it requires moving beyond the traditional messaging approach for evidence based intervention to address specific needs and gaps.

## Background

Over the last decades, the world has documented a remarkable success in the fight against malaria, especially in sub-Saharan Africa including Ethiopia [1, 2]. Nevertheless, malaria remains a global public health and socio-economic burden. In 2014, the World Health Organization (WHO) estimated that malaria caused 198 million illnesses worldwide leading to approximately 584 000 deaths. Africa is the most affected continent with 90 % of all malaria deaths mainly occur in children less than 5 years of age. In 2013, an estimated 437,000 African children died before their fifth birthday due to malaria [1]. Despite tremendous efforts and commitments, malaria continues to be one of the major health and socio-economic burdens in Ethiopia [2, 3]. In 2013, there were more than three million confirmed malaria consultations, recorded as the first cause of morbidity (11.7 %) and the third leading cause of health facility admission (7.8 %). Likewise, it was the fourth leading cause of health facility consultation (9.6 %) and the second leading cause of health facility admission among under five children. In the same year, malaria accounted for 5.8 % (both confirmed and clinical) death among under five children [3].

Ethiopia has given considerable attention to malaria prevention and control [4]. With sustained universal coverage of key malaria interventions, the country has envisioned eliminating malaria by 2020 [5]. As part of this strategic vision, the government has aimed to achieve malaria elimination in areas with historically low malaria transmission and achieve near zero malaria deaths in all other parts of the country by 2015 [5]. To this effect, the national malaria control program has planned to scale up the coverage and distribution of long-lasting insecticidal nets (LLINs) to 100 % and increase ownership (at least two LLINs per household) in malaria endemic areas, and reach 86 % LLIN use among pregnant women and under five children by 2015 [4, 5]. Given that this plan is in due date, the government and key malaria partners have updated their strategic plan to attain the long-term goal of worldwide malaria elimination and eventual eradication by 2040–2050. For instance, the President’s Malaria Initiative (PMI) Strategy (2015–2020) reaffirms continuing to sustain universal access and use of LLINs to assist PMI supported countries to progress towards elimination and eventual eradication [2].

To ensure universal access, WHO recommends that one LLIN should be distributed for every two people at risk of malaria, and thus, improving access to LLINs should be the first priority [6]. Evidence has shown that there is a high correlation between access and use of LLINs. However, in areas where LLIN use is lower, WHO recommends the roll-out of well-designed behavior-change communication (BCC) interventions [7]. For several years, two main indicators were being used to assess LLIN use; the “proportion of households owning at least one LLIN” and the “proportion of children under five and pregnant women sleeping under LLIN the previous night” [8]. However, these indicators have their own limitations since they consistently show a substantial gap between ownership and actual use of nets by vulnerable groups i.e. children and pregnant women or other family members. Although lack of access to LLIN contributes to non-use, behavior-driven failure also plays a key role [9].

In order to measure access to LLIN in a more appropriate way, the Roll Back Malaria (RBM) recommended two additional core LLIN indicators [10]: the “proportion of households with at least one LLIN for every two people” and the “proportion of the population that has access to an LLIN within the household” [11]. The first indicator is used in combination with the previous indicator “proportion of households with at least one LLIN” to estimate the ownership gap (i.e. households with no or insufficient LLIN) in a better way. The second indicator assists to measure the use gap which is due to behavioral failure. However, access to and use gaps of LLINs have been studied little in Ethiopia. Therefore, this study investigated LLIN access and use gaps among households in three districts of Jimma Zone, Southwestern Ethiopia.

## Methods

### Study setting

**Fig. 1:**
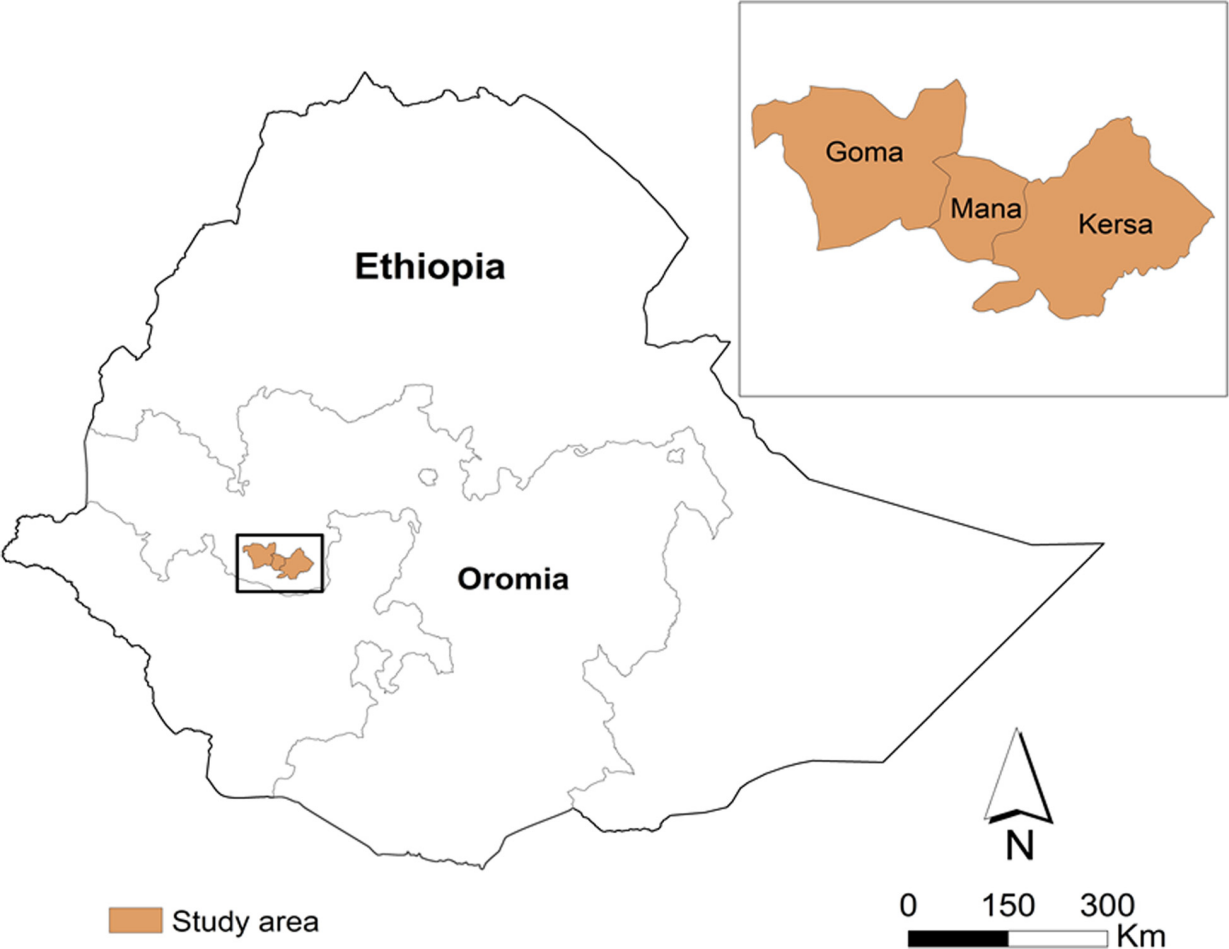
Map of the study area

The data were obtained from a larger community based cross-sectional survey conducted during December 2013 to January 2014 in three districts (Mana, Goma and Kersa) of Oromia region. The three districts are parts of Jimma Zone in Oromia. The Zone is located on the geographic coordinates of 7° 40′ 0″ N latitude and 36° 50′ 0″ E longitude in Oromia, Southwestern Ethiopia. Figure 1 shows map of the study area. The data were collected to establish baseline indicators for malaria education interventions implemented through school students and religious leaders. The three districts were purposively selected based on the burden of malaria and absence of ongoing malaria interventions by Non-Governmental Organizations. Goma district is situated at an altitude that ranges from 1380 to 1680 m, and about 20.1 % of the landmass of the district is swampy. The total population of this district was 213,023 and only 5.9 % of its population were urban dwellers. Mana is a district neighboring to Goma and has a total population of 146,675 where females accounts for 49.1 % and only 3.0 % of the population were residing in urban areas. The altitude of the district ranges from 1470 to 2610 m above sea level. Likewise, Kersa district is located at altitude that ranges from 1740 to 2660 m. The population of the district was about 165,391, of whom 49.5 % were females, and 3.28 % of the population were living in urban areas. Oromo is the dominant ethnic group and the majority of inhabitants were Muslims in the three districts [12]. The three districts are found in similar geographical and ecological conditions. And, malaria is an endemic in the locality. In Ethiopia, which is also true to the study area, malaria transmission is mostly unstable and seasonal. Peak transmission generally occurs between September and December after the main rainy season (i.e. from June to August). However, in southwestern of Ethiopia where the study area is located, rainfall season often begins earlier in April and the area lacks clearly defined rainfall season. In addition, following a short rainy season in February and March, some minor malaria transmission also occurs in April, May and June. The country also experiences some inter-annual variation in rainfall and temperature leading to a persistent risk of malaria transmission patterns [5, 13, 14]. Comprehensive data are lacking on the pattern of malaria in the study area but one community based study in the adjust district reported a prevalence of 10.5 % where Plasmodium falciparum accounted for 40.9 % [15], and another retrospective study in one of the study district reported that Plasmodium falciparum accounts for 62.4 % of malaria cases [16].

**Fig. 2:**
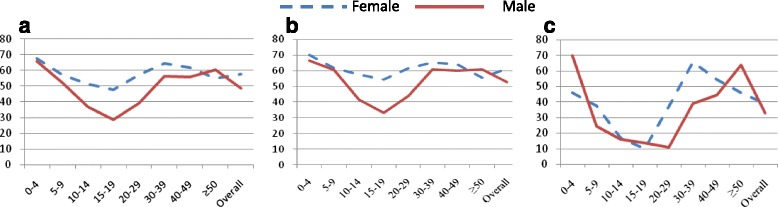
LLIN use among household members by age and sex, Jimma, Jan 2014. **a** shows LLIN use in households with at least one LLIN; **b** shows LLIN use in households with sufficient access and **c** indicates LLIN use in households with insufficient LLIN. Overall, except after the age of 48 years, the proportions of females who were using LLIN was higher than that of males in all age groups (**a** and **b**). Nevertheless, in households with insufficient access to LLIN, males were given priority in under five children implying that insufficient access might have caused gender disparity (**c**). On the other hand, LLIN use was very low among young people (age 15–19 years) which did not vary by household access to the LLIN. Qualitative data also confirmed that females and children were given priority among household members in general. However, insufficient access to LLIN led to gender disparity

### Study design and population

This baseline study employed a community cross-sectional design triangulated with qualitative methods to assess households’ behavioral practices on malaria prevention and control in the three districts. However, this article reported LLIN access and use gaps in the study community. The sample size was determined using single population proportion formula (*n* = Z_1-α/2_^2^ p (1-p)/ d^2^) assuming the proportion of under five children who slept under LLIN during the previous night (55.4 %) [17], 5 % marginal error, 95 % confidence interval, design effect of 2 and 10 % non-response rate. This gave a sample size of 834 households. The households were selected randomly from 13 Gandas (the smallest administrative unit in Oromia, Ethiopia). The number of Gandas was decided considering logistic feasibility and resource available for the study. In each district, Gandas with high malaria risk were randomly selected. The stratification of malaria transmission as high risk, in the study districts, generally followed the national guideline which considered elevations below 2000 m above sea level as high risk area [2, 5]. In addition, some local factors such as malaria case load and availability of water collections for vector breeding were considered to define Gandas as having risk of malaria transmission. Consequently, the number of Gandas with risk of malaria transmission were 20, 16 and 26 in Kersa, Mana and Gomma respectively. Eight Gandas were randomly selected from Kersa and Mana (four each) and five Gandas from Goma district. First, based on probability proportional to size, the sample size was allocated to each district and within district; the sample size was proportionally allocated to each Ganda based on household size. Then, systematic sampling method was employed to select households for an interview. Household heads were considered for interview. However, the spouse was interviewed if household head was not present at the time of visit. Given that urban population approximately accounts for 15 % in Ethiopia [17], to have fair representation, about 15 % of the households were selected from urban settings. For the purpose of this study, urban was defined as an administrative town with municipality service. A total of six (i.e. two in each district) Focus Group Discussions (FGDs) with women and men in separate groups (three in each) were conducted. In addition, 11 key informant interviews, eight in Mana and Goma (four in each) and three interviews in Kersa, were also conducted with various categories of health workers, school teachers and religious leaders, to complement the quantitative data.

### Indicators of LLIN coverage and access

Standard questionnaire adapted from literatures was used to collect data onLLINaccess, ownership, and coverage [17, 18]. Household and population based indicators were used for assessment of universal coverage of LLIN [8, 10, 11].

## Household based indicators

### Proportion of households in the study with at least one LLIN (P_1_)

The numerator consists of all households that own at least one insecticide mosquito net and the denominator is the total of number of sampled households.

### Proportion of households with at least one LLIN for every two people (P_2_)

The numerator contains all households where the ratio between numbers of LLIN owned, the number of household members is 0.5 or higher and the denominator is the total number of sampled households. This is the indicator of proportion of households having sufficient access to LLIN.

## Population-based indicators

### Proportion of population with access to LLIN within the household (P_3_)

The numerator includes all household members in the sample who had access to LLIN assuming each LLIN was used by two people and the denominator is the population in the sample. The calculation of the numerator was performed based on Roll Back Malaria Monitoring and Evaluation Reference Group’s (MERG) procedures [11]: first, potential LLIN users were obtained by multiplying the number of LLIN in each household by the factor of two. Second, the access indicator was calculated by dividing the potential LLIN users by the number of members of each household and computing the overall sample mean of that fraction.

### Proportion of population sleeping under an LLIN the previous night (P_4_)

The numerator contains all members of the household identified as one of the users of an LLIN based on the listings of member and the denominator is the population included in the sample.

Additionally, two indicators were computed according to MERG’s recommendation [11] in order to facilitate interpretation of ownership and use gaps. They include Proportion of households with at least one LLIN for every two people among households owning any LLIN (P_5_). This measures the saturation with LLIN for households with any LLIN. And, the inverse of P_5_ (1-P_5_) gives the intra-household ownership gap.

Proportion of population sleeping under an LLIN the previous night among those with access (P_6_). The fact that method of calculating the access indicator does not allow allocation of access to specific individuals within the household, P_6_ was calculated for the overall population included in the study by dividing the number of people who slept under LLIN the previous night to the total population who had access. The inverse of this indicator (1-P_6_) gives the use gap.

### Data collection

The quantitative data were collected by experienced and trained field enumerators. The tool was translated into Afan Oromo (local language) and pretested on 5 % of the sample size in similar setting. The whole process of data collection was closely supervised by three academic staffs of Jimma University. The FGDs and the key informant interviews were conducted by experienced public health professionals who had master’s degree of qualifications.

### Data analysis

The data were analyzed using SPSS version 17.0 software package. Proportions were computed for each indicator and presented in tables and figures. Binary logistic regression was applied to determine the association of background factors with LLIN use. A 95 % confidence interval and level of significance less than 0.05 were used during the analysis. The data from FGDs and the interviews were transcribed verbatim and then translated into English, and finally triangulated with quantitative findings.

### Ethical consideration

The study was approved by the research ethics committee of Jimma University (Ref: RPGC/260/2013). The purpose of the study was explained to each respondent and informed verbal consent was obtained from all respondents.

## Results

### Demographic characteristics of the respondents

A total of 798 households participated in the study giving a response rate of 95.7 % (Table 1). A total of 4107 persons were living in these households with average family size of 5.1 ± 2.1.

**Table 1:** Demographic characteristic of the respondents, Jimma, Jan 2014

Demographic characteristics		Frequency	Percent
Districts	Kersa	256	32.1
	Mana	218	27.4
	Goma	324	40.5
Residence	Urban	131	16.4
	Rural	667	83.6
Sex of respondents	Male	401	50.3
	Female	397	49.7
Marital status	Married	669	83.9
	Widowed	95	11.9
	Others^a^	34	4.2
Educational status	Cannot read and write	399	50.0
	Read and write	214	26.8
	Formal education	185	23.2
Religion	Muslim	640	80.2
	Orthodox	144	18.0
	Protestant	14	1.8
Ethnicity	Oromo	650	81.4
	Amhara	58	7.3
	Dawuro	49	6.1
	Others^b^	41	5.2
Occupation	Farmer	614	77.0
	Private job	84	10.5
	Government employ	51	6.4
	Merchant	49	6.1

^a^single, divorced, engaged ^b^keffa, Silte, Yam, Gurage

### LLIN ownership

A total of 1174 functional LLINs were accessed in 798 households with mean1.5 ± 1.3 LLINs per household. Less than half (44.9 %) of the nets were distributed to households very recently (i.e. during the last six months). Overall, 70.9 % (95 % CI: 67.8–74.1 %) of the study households had at least one LLIN at the time of the survey (Table 2). However, of the households with at least one LLIN, the coverage of at least one LLIN for every two people was 88.9 % (95 % CI: 86.3–91.4 %).

**Table 2:** LLIN ownership by selected background characteristics, Jimma, Jan 2014

Background characteristics		HHs at least with 1 LLIN (P_1_) % (95 % CI)	HHs with at least 1 LLIN for every 2 people (P_2_) % (95 % CI)	HHs with at least 1 LLIN for every 2 people if any LLIN % (P_5_) (95 % CI))	Number
District	Kersa	96.9 (94.7–99.0)	94.5 (91.7–97.2)	97.6 (95.7–99.5)	256
	Mana	79.8 (70.0–80.0)	66.5 (60.2–72.7)	83.3 (77.7–88.8)	218
	Goma	44.6 (39.2–50.0)	35.8 (30.5–41.0)	80.6 (74.1–87.0)	324
Residence	Urban	61.1 (52.7–69.5)	53.4 (44.8–61.9)	87.5 (80.2–94.7)	131
	Rural	72.9 (69.5–76.3)	64.9 (61.2–68.5)	89.1 (86.3–91.8)	667
Sex	Male	69.0 (64.5–73.6)	64.3 (59.5–69.0)	88.4 (84.7–92.0)	397
	Female	72.8 (68.4–77.2)	61.7 (56.9–66.4)	89.4 (85.7–93.04)	401
Education	Yes	71.0 (67.4–74.5)	62.8 (58.9–66.6)	88.5 (85.5–91.5)	613
	No	70.8 (64.2–77.4)	63.8 (56.8–70.7)	90.1 (84.9–95.2)	185
Overall		70.9 (67.8–74.1)	63.0 (59.6–66.3)	88.9 (86.3–91.4)	798

*HHs* Households

### Access to and use of LLIN

At the time of interview, only 491(41.9 %) of the LLINs were hung over the sleeping areas while more than half 641(54.6 %) of the nets were kept folded. Table 3 shows LLIN access and use by district and place of residence. Nearly half, 51.9 % (95 % CI: 50.5–53.5 %), of the study population (2130/4107) had access to LLIN within the households, with mean access 54.7 % (95 % CI: 51.9–57.5 %). LLIN access was the highest in Kersa district and the lowest in Goma. Likewise, the rate of access to LLIN was higher in rural than urban settings.

**Table 3:** Access and LLIN use by district and place of residence, Jimma, Jan 2014

Background characteristics		Proportion of people with access to LLIN (P_3_) % (95 % CI)	Mean % of population with access to an LLIN % (95 % CI)	LLIN use the previous Night(P_4_) % (95 % CI)	LLIN use the previous night if Access (P_6_) % (95 % CI)	Ratio of LLIN use to access (%)	Number
District	Kersa	79.0 (77.1–81.3)	83.5 (80.6–86.4)	57.8 (55.3–60.3)	73.1 (70.5–75.6)	69.2	1450
	Mana	51.4 (48.7–54.6)	57.2 (52.4–62.0)	36.0 (33.2–38.8)	70.1 (66.3–73.8)	62.9	1108
	Goma	26.8 (24.7–29.1)	30.4 (26.2–34.5)	21.9 (19.8–23.9)	81.9 (78.2–85.6)	72.0	1549
Residence	Urban	43.5 (39.7–47.3)	46.6 (39.5–53.6)	30.4 (26.9–33.9)	70.0 (64.7–75.27)	65.2	667
	Rural	53.5 (52.0–55.4)	56.4 (53.3–59.4)	39.9 (38.3–41.5)	74.7 (72.7–76.6)	70.7	3440
Overall		51.9 (50.5–53.5)	54.7 (51.9–57.5)	38.4 (36.9–39.9)	73.1 (71.2–74.9)	70.2	4107

Regarding LLIN use, 38.4 % (95 % CI:36.9–39.9 %) of the study participants slept under LLIN the previous night (Table 3). This net use coverage was higher in rural settings. Of those who had access to the LLIN, the proportion of people who used LLIN the previous night increased to 73.1 % (95 % CI:71.2–74.9 %) with little variations by district and place of residence. This gave an overall use to access ratio of 70.2 %. Despite low access to LLIN in Goma district, the prevalence of LLIN use was relatively higher among those who had access to it (Fig. 2).

### LLIN ownership and use gaps

This study identified two broad LLIN gaps (i.e. ownership and use gaps). Accordingly, 29.1 % (95 % CI:25.9–32.2 %) of the study households did not have LLIN at the time of the survey. Similarly, intra-household net gap was 11.1 % (95 % CI:8.5–13.6 %), meaning 11.1 % of the households with LLIN did not have sufficient access. Of those households who had any LLIN, 88.9 % (503/566) had sufficient LLIN for usual household members. However, 20.5 % (*n* =103) of these households had excess LLINs (i.e. more than one LLIN for every two people). On the other hand, of those population with sufficient access to LLIN, 26.9 % (95 % CI:25.0–28.7 %) did not actually use it. Table 4 shows reasons for not using LLIN.

**Table 4:** Barriers against LLIN use as identified through FGDs and key informant interviews

Characteristics of Nets, and Sleeping Arrangements	
•	Shape of the net: Given the rectangular nature of the nets, it is not comfortable to hang over sleeping area, ‘incompatibility with sleeping arrangements and house style’.
•	No bed: Some respondents mentioned people do not use LLIN if they don’t have bed or when their bed is under maintenance.
•	Sleeping outdoor: Many FGD and key informants mentioned that adolescents often sleep outdoor which often makes LLIN use difficult.
•	Perceived low efficacy of LLIN: Most participants argued that unlike past times, the net stopped killing mosquitoes and other insects and as result people throw it away or use for other purpose. *“When the chemical in the LLIN is unable to kill mosquito, people may use it for other purposes, as curtain or as cover of other materials.” [FGD participant]*
•	Fear of chemical: *“The chemicals in it [LLIN] sometimes cause irritation, cough and itching.”* [FGD participant]
Seasonality of Mosquitoes Bite	
•	No mosquito bite: Given the study was conducted during dry seasons, people argued that mosquito does not exist during dry season and there is no need to sleep under the net.
•	Saving LLIN for another time: People mentioned that they saved the nets for more risky time. This may confirm the fact that more than half of the nets were kept folded at the time of visit for interview.
Accessibility factors	
•	Insufficient access to LLIN within households.
•	Mal-distribution of LLIN that leads to either scarcity or excess nets
Personal factors	
•	Lack of awareness and carelessness among families
•	Using the nets for other unintended purposes, as ‘mattress’, ‘to cover toilet’
•	Low perceived risk of malaria infections
•	Throwing away once it is ‘thorn out ‘and/or ‘becomes dirty’

#### Association of demographic factors with LLIN use

Table 5 shows results regarding the association of background factors with LLIN use among household members with full and insufficient access to LLIN. Of household members with full access to LLIN, the use was significantly lower in Mana district (AOR = 0.56; 95 % CI: 0.46–0.68, *P* = 0.001). Females were 1.52 times more likely to use LLIN compared to males (AOR = 1.52; 95 % CI:1.25–1.83, *P* = 0.001). On the other hand, children aged 0–4 years (AOR = 2.28; 95 % CI:1.47–3.53, *P* = 0.001) and 5–9 years (AOR = 1.70; 95 % CI:1.11–2.61, *p* = 0.014) were more likely to use LLIN compared to older people (age ≥50 years) and other household members. Meanwhile, of the households with insufficient access to LLIN, the use was significantly lower among urban residents (AOR = 0.37; 95 % CI: 0.18–0.76, *p* = 0.007). In these households, children aged 0–4 years were more likely to use LLIN as compared to older people (AOR = 7.22; 95 % CI:1.74–29.88, *p* = 0.006). Nevertheless, the LLIN use was significantly lower for younger son/daughter and other family members as compared to heads of households (Table 5).

**Table 5:** Association of demographic characteristics with LLIN use the previous night, Jimma, Jan 2014

Background characteristics	HHs with sufficient access to LLIN				HHs with insufficient access to LLIN		
	LLIN use the previous night				LLIN use the previous night		
		Yes n (%)	No n (%)	AOR (95 % CI)	Yes n (%)	No n (%)	AOR (95 % CI)
District	Kersa	821 (62.0)	504 (38.0)	1	17 (36.2)	30 (63.8)	1
	Mana	322 (47.9)	350 (52.1)	0.56 (0.46–0.68)*	77 (41.2)	110 (58.8)	1.36 (0.63–2.94)
	Goma	285 (57.3)	212 (42.7)	0.82 (0.66–1.01)	55 (30.1)	128 (69.9)	0.68 (0.31–1.50)
Residence	Urban	185 (57.5)	137 (42.5)	0.99 (0.78–1.27)	18 (28.1)	46 (71.9)	0.37 (0.18–0.76)*
	Rural	1243 (57.2)	929 (42.8)	1	131 (37.1)	222 (62.9)	1
Sex	Female	783 (61.3)	494 (38.7)	1.52 (1.25–1.83)	75 (38.9)	118 (61.1)	0.98 (0.54–1.76)
	Male	645 (53.0)	572 (47.0)	1	74(33.0)	150 (67.0)	1
Age	0–4	220 (68.3)	102 (31.7)	2.28(1.47–3.53)*	27 (62.8)	16 (37.2)	7.22 (1.74–29.88)*
	5–9	232 (61.1)	148 (38.9)	1.70(1.11–2.61)	26 (31.7)	56 (68.3)	1.69 (0.43–6.53)
	10–14	195 (49.4)	200 (50.6)	1.02 (0.67–1.56)	11 (16.4)	56 (83.6)	0.68(0.16–2.82)
	15–19	118 (44.0)	150 (56.0)	0.83 (0.53–1.28)	6 (12.0)	44 (88.0)	0.45 (0.09–2.19)
	20–29	212 (54.1)	180 (45.9)	1.02 (0.72–1.46)	13 (23.6)	42 (76.4)	0.52 (0.15–1.83)
	30–39	170 (63.2)	99 (36.8)	1.20(0.84–1.71)	30 (56.6)	23 (43.4)	0.69(0.25–1.87)
	40–49	117 (62.2)	71 (37.8)	1.17(0.79–1.73)	14(48.3)	15 (51.7)	0.42 (0.14–1.24)
	≥50	164 (58.6)	116 (41.4)	1	22 (57.9)	16 (42.1)	1
Relationship to household head	Head	276 (62.3)	167 (37.7)	1	32 (54.2)	27 (45.8)	1
	Spouse	278 (63.3)	161 (36.7)	0.74 (0.53–1.04)	39 (66.1)	20 (33.9)	1.87 (0.68–5.18)
	Son/daughter	794 (55.9)	627 (44.1)	0.54 (0.38–0.77)*	74 (27.2)	198 (72.8)	0.14 (0.04–0.48)*
	Others	80 (41.9)	111 (58.1)	0.32(0.21–0.50)*	4 (14.8)	23 (85.2)	0.08 (0.02–0.35)

*Significant at *p* < 0.05

## Discussion

This study measured LLIN ownership, access and use gaps in malaria endemic settings in Southwestern Ethiopia, based on MERG indicators [10, 11]. It reported LLIN ownership prevalence of 70.9 % which is a better coverage compared to previous national and regional reports [17]. Nevertheless, the result was far behind the national target (100 % coverage) in malarious areas of Ethiopia by 2015 [5]. Even though this finding is nearly similar with some previous reports [19–21], there were many local studies that reported higher LLIN ownership coverage [15, 22, 23]. In one study, 77 % of the households had at least one LLIN [15]; another study reported 85.5 % [22]; and greater than 90 % ownership coverage was also reported elsewhere [23, 24]. In fact, two of the study districts in this study (Kersa and Mana) had quite higher LLIN ownership coverage which could be due to recent distribution of LLIN. On the other hand, LLIN ownership coverage was relatively lower in urban settings (61.1 % versus72.9 %) which could be due to limited and inappropriate LLIN distribution campaign in urban areas. This phenomenon could also be related to population dynamics in urban settings. Evidence has also shown that in many African countries LLIN distribution campaigns usually focus on rural settings [25] which might lead to lower LLIN coverage in urban areas. Thus, given the global recommendations to achieve universal LLIN coverage [6], particularly in malaria endemic settings, it is crucial to promote LLIN distribution strategies that fit into to urban contexts.

The ownership coverage indicator is basically relevant to measure the geographical distribution of the nets though it is with limited information about intra-household LLIN saturation which provides crucial information to roll out behavioral driven gaps [7]. WHO recommends universal coverage assuming one net for every two people and this goal remains the intended outcome for all areas at risk of malaria [6, 26].

Considering one LLIN for every two people, only 63.0 % of the sampled households in this study had enough LLIN for every member of the household. However, analysis of those households who owned any LLIN showed that ownership of at least one LLIN for every two people was increased to 88.9 % with intra-household net gap of 11.1 %. In contrast, a significant numbers of extra LLINs were noted in large portion of households (20.5 %) suggesting inequitable distribution of LLINs in the community. Households might have saved some of the nets for times of high transmission season or due to fear of shortage of nets which could be accounted for the availability of extra LLIN in some households. In addition, some households might have purchased more nets or there could be a possibility of routine distribution of LLIN through antenatal and immunization programs. This phenomenon was also reported in the qualitative part of this study. Some earlier studies also reported availability of extra LLINs in some households while other families were without any LLIN [22, 27].

Another important population based LLIN indicator is the proportion of population with access to LLIN within the household assuming each LLIN was used by two people. Consequently, despite fairly high coverage of ownership of at least one LLIN, nearly half of the population had no access to LLINs. This shows that there a huge access gap in these malaria endemic settings contrary to the WHO universal access coverage and national target [5, 6, 10].

In line with global malaria control and elimination efforts, Ethiopia has planned for malaria elimination which demands achieving and sustaining universal coverage of at least 80 % of all malaria interventions coverage including LLIN usage. This is necessary to move away from scaling-up for impact (SUFI) to sustained control and pre-elimination phase [10]. However, based on the findings, the progress towards the national target could be very slow as the current coverage is far from the target set to be achieved by 2015 [10]. In order to achieve sustainable coverage of LLIN, WHO recommends that countries should apply a combination of mass free distributions and continuous distributions through multiple channels, particularly through antenatal and immunization programs [28].

Use of LLINs is one of the most cost-effective interventions, and high use rates are a central goal in malaria control programs in malaria endemic settings [7, 8, 10, 28]. However, in this study, only 38.4 % of the household members slept under LLIN the night before the survey. The utilization rate was critically low in Goma district where only 21.9 % of the people reported sleeping under LLIN the previous night. The lack of access to LLIN could account for the low utilization rate in this district. The fact that more than half of the LLINs were kept unused at the time of the visit could also justify the low LLIN use coverage in this community which is also documented in earlier study [15]. Low risk perceptions, lack of access, negligence, saving and misusing nets, technical difficulties related to hanging, and perceived degraded efficacy of the nets were also cited reasons for poor utilization of LLIN. Nevertheless, consistent with previous reports from Ethiopia [17, 18], sleeping under LLIN was relatively higher among pregnant women and under five children. In contrast, it is lower compared to the results from some earlier studies [15–24, 27, 29–32]. Lower LLIN usage in the current study could be attributed to the time of the study (dry season) in which malaria transmission is low and people might not use LLIN. This phenomenon was also noted in the qualitative investigations in which several participants mentioned that people were less concerned about malaria during dry season when people are less likely to use mosquito nets. In unstable malaria transmission contexts, such as in the current study, this finding has important implications for malaria program in that it signifies the need to promote continued usage of LLIN. On the other hand, the deterioration of LLIN in both quality of insecticide and physical integrity might also be negatively impacted on LLIN use and its effectiveness. Even though evidence from the qualitative part of the study provided some insight about the converse relationship between perceived quality of LLIN and its use, this study did not provide in-depth information on this issue, and more studies could be important to gain further understanding about it.

Excluding households who did not own any LLIN from analysis, previous night LLIN use increased to 52.9 % among the study households with at least one LLIN and to 73. One percent among households with sufficient access with overall ratio of use to access being 70.2 %. This ratio allowed identifying the difference between non-use due to lack of access and non-use due to behavioral failure. Hence, a significant portion of the population who had access to LLIN did not actually use it, showing a substantial gap between access and actual use. Behavioral failure was quite high considering the general expectation that access led to higher user coverage [33]. Nevertheless, despite low LLIN access rate in Goma district, there was a strong habit of sleeping under LLIN when there was access to LLIN. This entails non-use was mostly linked to lack of nets than behavioral failure in this district. This fact may indicate existence of good awareness about malaria and LLIN use in this district. With regard to gender, females were more likely to use LLIN than males across all age groups in households with sufficient access to LLIN. Moreover, under five children were also more likely to use LLIN than younger adults and older people. This could reflect the success of the conventional communication approach that encourages LLIN use by women and children. Of course, it would be a logical argument as women often sleep under the same net with children.

The rate of net use was consistently lower among people in the age group of 10–19 years whether the households had sufficient access or not, which is also documented in some previous studies [32, 34, 35]. The fact that LLIN use in this age group did not depend on LLIN access reflects existence of huge behavioral failure in this specific age group calls for targeted behavioral change communication interventions. Results from the qualitative component of the study also complemented this finding in that older children (usually aged 10–20 years) often sleep outdoor which makes LLIN use less likely. Some recent reports revealed that the indoor biting behaviors of mosquitoes have been shifting to outdoor, and outdoor sleeping significantly increases the risk of malaria infection [36, 37]. The outdoor sleeping practice could be a potential challenge to universal LLIN usage which in turn increases residual transmission to be maintained in the community despite high indoor LLIN utilization. This requires reorientation of the conventional LLIN promotion approaches which focused mostly on indoor interventions with particular attention to women and children.

In households with insufficient access to LLIN, children were more likely to sleep under LLIN although males were given priority for LLIN use. This implies that insufficient access to the LLIN might have led to gender difference in LLIN use which was also cited in another study [38]. Perhaps, gender-specific behavior change communication intervention strategies could help to promote gender equity. It is also important to look at specific population segments and groups when designing and implementing a behavior change intervention strategy for LLIN promotion.

Evidence has shown that BCC plays a vital role in creating demand and increasing the LLIN use; makes families use their nets regularly and care and repair them [39]. Furthermore, BCC continues to play a key role even when the risk of malaria infections is greatly reduced. BCC can serve as a tool in malaria elimination efforts by targeting hot-spot reservoirs of infection which often overlooked during planning in control programs [39, 40]. Certainly, the prospect of achieving and sustaining universal user coverage of LLIN in malaria endemic areas heavily depends on a solid investment and well-designed BCC interventions.

### Limitations of the study

Since the study was conducted during low malaria transmission season, the finding may not reflect year round situations of LLIN use among the populations. Furthermore, this study was limited to a small geographical area with similar seasonal and unstable malaria transmission and it may not reflect LLIN use in other eco-epidemiologic settings in Ethiopia.

## Conclusions

Ownership coverage of LLINs was lagging behind the national and global targets in the study community, characterized by lower access rate with a relatively wide intra-household net coverage gap. There was a wide gap between ownership of nets and actual use. However, higher access rate was basically associated with increased net use although not to the expected level. It was noted that low access to LLIN leads to gender disparity particularly among children. On the other hand, behavior-driven non-use of LLIN and non-use due to lack of the nets remained significant constraints to universal usage of nets in the study setting. Given that LLIN access to and use coverage was far short of the targets, it conveys two important messages to the malaria control program for speedy move towards elimination and eventual eradication: 1) achieving and maintaining universal LLIN coverage is crucial, 2) addressing behavior-driven non-use through well designed and tailored behavioral change interventions. Further, it calls for the need to go beyond the traditional messaging approach to evidence based practice by taking into account gender and age specific tailored interventions. Finally, it should be noted that this study provided cross-sectional evidence on LLIN access and use which is useful information to inform LLIN distribution and usage programming. It also indicates the need to conduct large scale study to model the variations in LLIN access and usage gaps.

## Abbreviations

BCC: behavior change communication
FGDs: focus group discussions
HEWs: health extension workers
IRS: indoor residual spray
LLINs: long-lasting insecticidal nets
MERG: malaria evaluation reference group
PMI: president’s malaria initiative
RBM: roll back malaria
WHO: World Health Organization
